# Arbuscular Mycorrhizal Fungi Response on Soil Phosphorus Utilization and Enzymes Activities in Aerobic Rice under Phosphorus-Deficient Conditions

**DOI:** 10.3390/life13051118

**Published:** 2023-04-30

**Authors:** Debasis Mitra, Periyasamy Panneerselvam, Ansuman Senapati, Parameswaran Chidambaranathan, Amaresh Kumar Nayak, Pradeep Kumar Das Mohapatra

**Affiliations:** 1 Department of Microbiology, Raiganj University, Raiganj 733134, West Bengal, India; 2 ICAR—National Rice Research Institute, Cuttack 753006, Odisha, India

**Keywords:** arbuscular mycorrhizal fungi, aerobic rice, soil enzymes, phosphorus utilization, P-deficient, plant growth promotion

## Abstract

The prominence of arbuscular mycorrhizal fungi (AMF) in sustainable rice production has long been recognized. However, there is little information about AMF response in aerobic rice cultivation under phosphorus (P)-deficient conditions. The aim of this experiment was to compare and determine the preeminent AMF effects on rice mycorrhizal colonization, responsiveness, P utilization, and different growth-promoting traits under P-deficient conditions. Different AMF genera *viz*. (*Funneliformis* sp., *Rhizophagus* sp., *Glomus* sp., *Acaulospora* sp., and *Claroideoglomus* sp.) in four different aerobic rice varieties developed by ICAR-NRRI, India (CR Dhan 201, CR Dhan 204, CR Dhan 205, and CR Dhan 207) were investigated using the check P-susceptible variety (IR 36) and the P-tolerant variety (Kasalath IC459373). Data analyzed through linear modeling approaches and bivariate associations found that AMF colonization was highly correlated with soil enzymes, particularly fluorescein diacetate (FDA) and plant P uptake. The microbial biomass carbon (MBC) and FDA content were significantly changed among rice varieties treated with AMF compared to uninoculated control. Out of four different rice varieties, CR Dhan 207 inoculated with AMF showed higher plant P uptake compared to other varieties. In all the rice varieties, AMF colonization had higher correlation coefficients with soil enzymes (FDA), MBC, and plant P uptake than uninoculated control. The present study indicates that AMF intervention in aerobic rice cultivation under P-deficient conditions significantly increased plant P uptake, soil enzymes activities and plant growth promotion. Thus, the information gathered from this study will help us to develop a viable AMF package for sustainable aerobic rice cultivation.

## 1. Introduction

Rice (*Oryza sativa* L.) is a major agricultural crop and staple food that feeds more than half of the world’s population, is grown in >100 countries with 90% of the total global production coming from Asia [1]. In India, rice is cultivated in an area of 45 million hectares and contributes to a great extent to national food security. Additionally, Asia alone consumes 90% of the freshwater diverted to agriculture in the entire world [2,3]. This will soon be a burden on the ecological balance in many areas, leading to water scarcity. In this case, aerobic rice cultivation is a modern practice for cultivating rice crops with durable water soil and suited, high-yielding varieties that are sown directly dry [4]. This approach saves water significantly; in China, the aerobic rice system of cultivation used 55–56% less water as compared to the traditional transplanted system of cultivation with water productivity that is 1.6–1.9 times higher [5]. To keep pace with the changing scenario, an estimated 22 varieties and 2 hybrids have been released for aerobic conditions in India [4]. According to Ghasal et al. [6], dry and aerobic soil can reduce the natural supply of phosphorus (P), making the application of P fertilizer more crucial for rice grown aerobically. P is necessary for all living organisms, and is a crucial nutrient for the expansion and development of the plants [7,8,9]. Phosphorus makes up about 0.2% of a plant’s dry biomass and is mostly present in tissue components such as phospholipids, nucleic acids, and adenosine triphosphate (ATP) [10]. P is the second most limiting nutrient after nitrogen (N) [11]. It may decrease agricultural productivity and slow down plant growth and development. P exists in three different forms in the soil: organic P, soluble inorganic P, and insoluble inorganic P [7]. The amount of total soil phosphorus varies between 30 and 65% in organic forms, which are unavailable to plants, and 35 to 70% in inorganic forms [12]. Organic P can be found in soil microorganisms and dead plants and animals. P becomes unavailable in the soil because of fixation and immobilization, and 70–90% of phosphate fertilizers become fixed in the ground [13,14]. Soil microorganisms, mainly arbuscular mycorrhizal fungi (AMF), play a key role in mobilizing phosphorus from the soil into plant-available forms [15,16,17,18]. In the root cortical cells of their host plants, AMF create highly branching fungal structures called arbuscules, with which they exchange inorganic minerals, particularly phosphorus, and carbon molecules. AMF are one of the most prevalent organisms in the mycorrhizosphere [19,20] and have interactions and colonization with more than 200,000 different species of host plants with more than 240 different AMF morphotypes [21]. The exploration of mycorrhizal symbiosis is one of the most promising methods for creating resource-efficient and resilient agricultural systems [20,22]. Several studies have reported AMF diversities in rice [23,24,25], but very limited information is available on their performance in aerobics under P-deficient conditions [26]. Additionally, some studies indicated that AMF have a host preference [27] and their performance will vary depending on different agroecosystems [28]. In aerobic rice cultivation, soil P fixation is one of the major problems which causes P deficiency in the soil resulting in yield reduction. The main idea of this study is whether the intervention of suitable AMF will resolve the issue of soil P deficiency in aerobic rice cultivation. Hence, the present study was conducted to evaluate the effect of AMF on P uptake and growth promotion in popular aerobic rice varieties under P-deficient conditions.

## 2. Materials and Methods

### 2.1. Low P Soil Sampling, Site Description, and AMF Inoculum and Propagation of AMF

**Figure 1:**
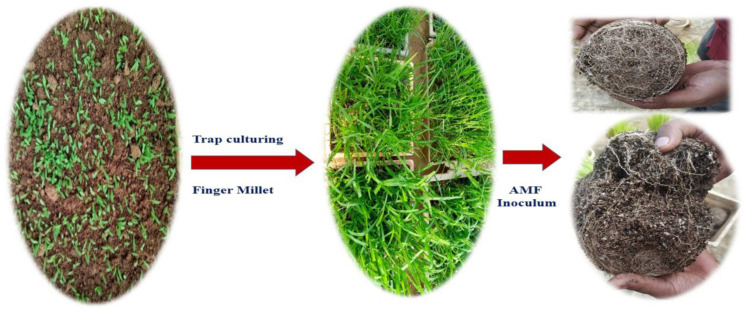
Monospecific AMF spore propagation using trap cultures and finger millet as host plant.

Low-phosphorus (P) soil was collected from Krishi Vigyan Kendra (KVK), Santhpur, ICAR—NRRI, Cuttack, Odisha (20°27′45.08″ N; 85°52′58.76″ E) for the experiment and analysis. The initial properties of the experimental soil were analyzed (Table 1). The sterilized soil was used for a pot experiment. The soil-based single AMF inoculum *viz*. *Funneliformis* sp., *Rhizophagus* sp., *Glomus* sp., *Acaulospora* sp. and *Claroideoglomus* sp. received from Microbiology, ICAR—the National Rice Research Institute (ICAR-NRRI), India, were used in this experiment together with inoculum containing 115–120 AMF spores/g of soil, which was multiplied using finger millet (*Eleusine coracana*) as the host plant in sterile soil using a trap culturing method (Figure 1) [27,29].

### 2.2. Experimental Site and Pot Experiment

The experiment was conducted during the 2020–2021 *Rabi* season (the Indian cropping season starting from the onset of winter from October-November until spring in March–April) in a controlled net house condition at Microbiology, the ICAR-National Rice Research Institute (NRRI), Cuttack, Odisha (latitude—20°25′ N, longitude—85°55′ E with an altitude of 24 m above mean sea level). The pot (5 kg) experiment was conducted with five different species of AMF and six rice varieties with three replications. The treatment details are as follows, T0: Control, T1: *Funneliformis* sp., T2: *Rhizophagus* sp., T3: *Glomus* sp., T4: *Acaulospora* sp. and T5: *Claroideoglomus* sp. In this experiment, four aerobic rice varieties *viz*. V1: CR Dhan 201, V2: CR Dhan 204, V3: CR Dhan 205, V4: CR Dhan 207 (CR Dhan 201, 204, 205, and 207 developed by ICAR-NRRI, Cuttack), and two check varieties *viz*. V5: IR 36 (P-susceptible) and V6: Kasalath IC459373 (P-tolerant) were used, and were collected from the Crop Improvement Division, ICAR-NRRI, Cuttack, India. After germination, three plants per pot were maintained. Soil (completely homogenized and transported to the laboratory in a cool pack) and all the plant samples from each pot were collected from all treatment after 60 days in order to estimate the AMF colonization, growth parameters (root length, shoot length, leaf area, chlorophyll, fresh and dry biomass), P uptake, soil chemical, microbial and enzymatic activities analysis [30].

### 2.3. Assessment of AMF Colonization and Spore Count

The method developed by Phillip and Hayman [31] was used to evaluate the rice root colonization of AMF [32]. Freshly collected root samples were gently washed to remove soil that was attached to the root surfaces, submerged in 10% potassium hydroxide (KOH) solution, and autoclaved for 15 min at 121 °C. The KOH solution was decanted, and the treated roots were rinsed with tap water three times until no brown colour appeared in the rinsed water. The treated root samples were further immersed in 2% hydrochloric acid (HCl) solution for 5 min. Without being rinsed with water, HCl was decanted, and the root samples were stained with 0.05% trypan blue (HiMedia, Maharashtra, India) in lacto-glycerol (400 mL lactic acid + 400 mL glycerol + 100 mL water) and autoclaved for 15 min at 121 °C. After autoclaving, the stained solution was decanted, and the roots were de-stained with lacto-glycerol solution to remove the excess stains and used for microscopic observations. The slide was prepared by keeping 10 segments of the stained root on a clean glass slide and observed under a compound microscope (Zeiss Stemi 508, Oberkochen, Germany). The method described by McGonigle et al. [33] was used to calculate the percentage of root colonization.

AMF root colonization was calculated using the formula:

*% of colonization* = *no. of root segments colonized* ÷ *total no. of root segments* × 100

### 2.4. Phosphorus Estimation in Plant Sample

Collected plant samples were dried in a hot air oven maintained at 60 °C for up to 5 days in order to attain a constant weight. The determination of P concentration in the plant sample was carried out using the vanadomolybdophosphoric acid method with a spectrophotometer [30,34]. A quantity of 1 gm of the dried plant sample and 10 mL of the concentrate HNO_3_ were added and kept overnight, following which 10 mL of tri-acid (HNO_3_, H_2_SO_4_, HCLO_4_ in a ratio of 9:4:1), was added and mixed properly. The mixture was kept in a hot plate at 100 °C for 1 h under a temperature rise up to 200 °C until the content reduced to 2–3 mL and turned colourless. The content was cooled and 10 mL of diluted HCL was added and filtered through Whatman No. 42. The filtrate volume was made up to 100 mL with distilled water. A quantity of 5 mL of the digested sample was taken and 10 mL of vanadomolybdate reagent was added (Merck, Darmstadt, Germany) and kept for 30 min. The absorption of the sample was measured at 420 nm with a spectrophotometer (Analytikjena specord-200, Jena, Germany). A standard curve was prepared with a phosphate solution (0.2195 gm of KH_2_PO_4_ in 500 mL of distilled water + 25 mL of 7N H_2_SO_4_ and made up to 1 L) and the P content of the plant sample was calculated from the standard curve.

### 2.5. Estimation of Soil Chemical, Enzymatic and Microbial Properties

The activity of the acid (AcP) and alkaline (AkP) phosphatase of soil samples was estimated by the method of Tabatabai and Bremner [35], using p-nitrophenyl as a substrate and expressed in l g of p-nitrophenyl phosphate (*p*NP) released per gram of soil per hour. Soil fluorescein diacetate activity (FDA) measurement was carried out by using Scherer and Ross [36] as modified by Adam and Duncan [37]. The concentration of fluorescein released during the assay was calculated using the calibrating graph produced from the 0–5 µg fluorescein mL^−1^ standard and expressed as µg fluorescein h^−1^g^−1^ soil [27]. Dehydrogenase activity (DHA) was estimated by the method of Casida et al. [38]), using triphenyltetrazolium chloride (TTC) as a substrate. Microbial biomass carbon (MBC) was determined using the chloroform fumigation extraction (CFE) method [39].

### 2.6. Statistical Analysis

The R version 4.2.2 [40] was used for statistical computing. For the identification of important variables related to AMF colonization in plants, a stepwise regression model was constructed using the “stepAIC” function available in the MASS package [41]. The Pearson correlation was constructed using the “ggpairs” function available in the GGally package [42].

## 3. Results and Discussion

Rice crops are very sensitive to water stress and reduction in water inputs with a consequent decline in yield [43]. Approximately 75% of the rice is produced by a conventional flooding method, and 3000–5000 L of water is needed to produce 1 kg of grains [4,44]. Researchers have developed several technologies to reduce water inputs in rice such as alternate wetting and drying, raised bed rice cultivation, saturated soil culture, a system of rice intensification, ground cover systems, and raised bed systems [45]. Some of the modern technologies additionally require puddling and ponded water during crop growth. In rice cultivation, the aerobic rice has been introduced to minimize the use of water, which is one of the promising water-saving technologies in rice production [46,47]. Aerobic rice reduces water use by 27–51% by limiting water loss due to seepage, percolation, and evaporation and increases water productivity by 32–88% [48]. It has been well documented that microorganisms enhance plant growth under abiotic stress [49].

### 3.1. Seed Germination of Rice Varieties

**Figure 2:**
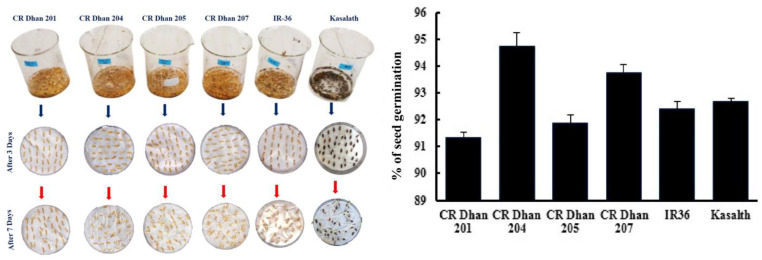
Percentage of seed germination of six rice varieties.

The seed germination percentages of four different aerobic rice varieties (CR Dhan 201, CR Dhan 204, CR Dhan 205, and CR Dhan 207), as well as another P-susceptible variety (IR 36) and P-tolerant variety (Kasalath IC459373) are given in Figure 2. CR Dhan 204 and 207 rice varieties showed the highest germination percentages. However, all the rice types had germination rates of >90%.

### 3.2. AMF Root Colonization in Different Aerobic Rice Varieties

**Figure 3:**
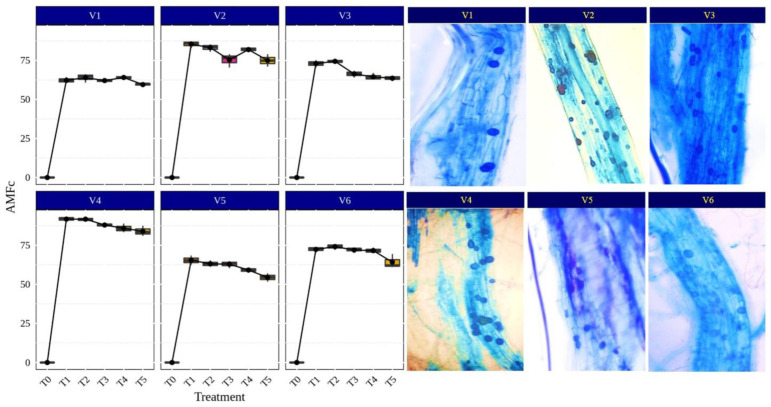
Percentage of AMF colonization (AMFc) in different rice varieties. Abbreviation: percentage of AMF colonization (AMFc).

AMF symbiosis increases nutrient and water uptake in plants by external hyphae, regulation of stomatal conductance and the increased activity of antioxidant enzymes. Under aerobic conditions, rice plants readily form mycorrhizal associations as compared to submerged conditions where the anoxic environment limits the mycorrhizal infection process [50,51]. Rice can also be grown with alternate irrigation to reduce the water input and to create aerobic conditions for better AMF fungi colonization in rice roots. Therefore, an investigation was undertaken to understand the benefits of AMF association for rice plant growth and development under aerobic conditions. Narwal et al. [44] found a 20% increase in the plant biomass and 58% higher colonization of *Glomus intraradices* and *G. mosseae* (currently *Funneliformis mosseae*) in upland rice varieties (Pyari, Satyabhama, CR Dhan 205 and CR Dhan 202) compared to lowland rice varieties (Pusa Basmati (PB) 1509, PB 1121, Pusa Sugandha 5 and PB 1612) in pot experiments with sterile soil. The AM plants enhanced the activities of glutamine synthetase and nitrate reductase; the rice genotypes with higher nitrate reductase and glutamine synthetase (Pyari and Satyabhama) also exhibited more (20%) biomass production and plant N content by 36% [44]. In our study, the results of the different AMF-inoculated rice varieties and its root colonization, presented in Figure 3, indicated that *Funneliformis* sp., *Rhizophagus* sp. and *Glomus* sp. showed higher colonization in CR Dhan 207 (91.75, 91.72 and 87.97%, respectively) and CR Dhan 204 (85.43, 83.19, and 75.37%, respectively), while the other genera of AMF recorded a root colonization in the range of 54.38–74.98%.

### 3.3. Effect of AMF Inoculation on Physiological and Agronomic Properties

**Figure 4:**
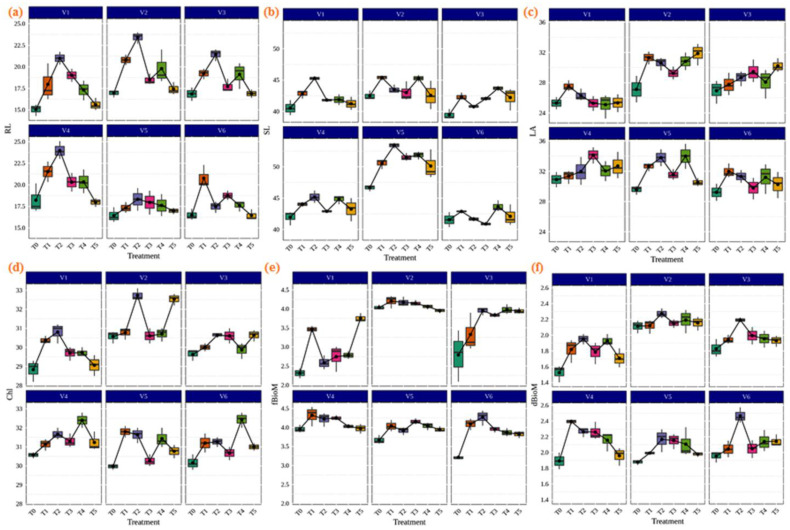
Enhancement of plant growth parameters due to AMF inoculation in different aerobic rice varieties. Abbreviations: (**a**) root length in cm. (RL); (**b**) shoot length in cm. (SL); (**c**) leaf area m^2^ (LA); (**d**) chlorophyll SPAD (Chl); (**e**) fresh biomass in gm. (fBioM); (**f**) dry biomass in gm. (dBioM).

Inoculation of AMF played an important role in the improvement of the biomass chlorophyll contents and physiological and agronomic parameters of the plant. It is widely believed that the inoculation of AMF provides the highest efficiency to host plants for plant growth. As shown in Figure 4, our results demonstrated that AMF inoculation in different rice varieties significantly increased the agronomic parameters, including root length (cm), shoot length (cm), leaf area (m^2^), chlorophyll (SPAD), fresh biomass (gm), and dry biomass (gm) compared to the control. The highest shoot and root lengths were found in IR36 (53.40 cm) and CR Dhan 207 (23.973 cm) with the treatment of *Rhizophagus* sp. (Figure 4a,b). In the rice variety CR Dhan 207 (34.127 m^2^), treatment with *Glomus* sp. showed the best improvements for the leaf area (Figure 4c). Chlorophyll (SPAD) levels were highest in CR Dhan 204 (32.73) with *Rhizophagus* sp.; CR Dhan 204 (32.53) with *Claroideoglomus* sp.; Kasalath IC459373 (32.43) and CR Dhan 207 (32.40) with *Acaulospora* sp. treatment (Figure 4d). The *Funneliformis* sp. treated with CR Dhan 207 (4.32 gm) and *Rhizophagus* sp. treated with Kasalath IC459373 (2.466 gm) had the maximum performance in terms of plant fresh biomass and dried biomass, respectively (Figure 4e,f). However, the plant growth parameters viz. root length, leaf area, chlorophyll and plant biomass showed themselves to be significantly higher in CR Dhan 207 and CR Dhan 204 inoculated with *Rhizophagus* sp., *Glomus* sp., *Funneliformis* sp., and *Acaulospora* sp.

### 3.4. Effects of AMF on Uptake of Plant P

**Figure 5:**
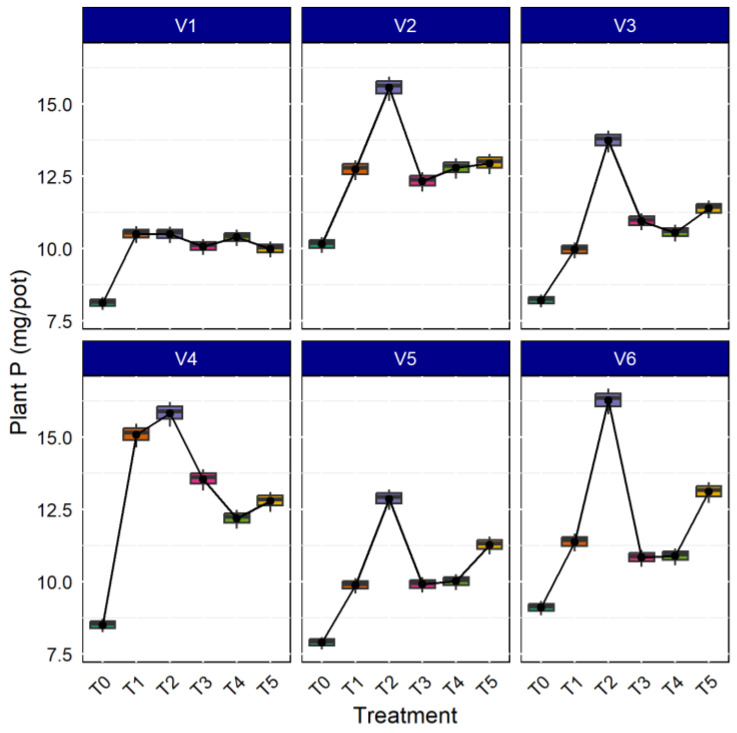
AMF inoculation on uptake of plant P in different aerobic rice varieties.

AMF both in aerobic and anaerobic rice cultivation increases nutrient concentration in the rice plant tissue; the bioavailability of nutrients increased in the soil solution due to mycorrhizae inoculation [52]. As shown in Figure 5, the P concentration in plants was higher in the rice variety CR Dhan 207 (14.796 mg. pot^−1^), followed by Kasalath IC459373 (14.186 mg. pot^−1^) and CR Dhan 204 (14.156 mg. pot^−1^). Additionally, all the rice varieties inoculated with *Rhizophagus* sp., showed maximum P uptake, followed by *Funneliformis* sp., and *Glomus* sp. inoculation. The results deciphered 16.60–28.50% higher P uptake with AMF inoculation in all the rice varieties, compared to the uninoculated control.

### 3.5. Responses of AMF on Soil Enzyme and Microbial Properties

**Figure 6:**
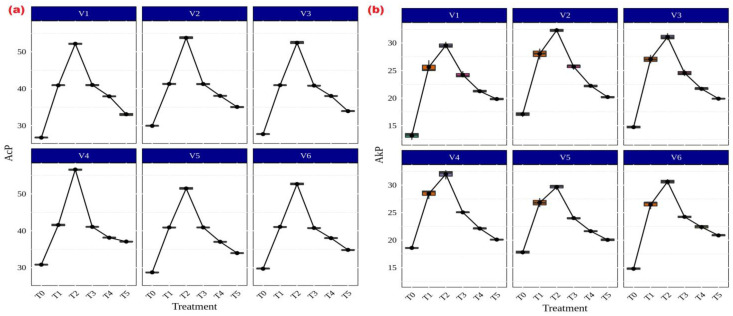
Enhancement of acid and alkaline phosphatase activities in different aerobic rice varieties. Abbreviations: (**a**) acid phosphatase (AcP) [µg p-nitrophenol released h^−1^ g^−1^ soil]; (**b**) alkaline phosphatase (AkP) [µg p-nitrophenol released h^−1^ g^−1^ soil].

Among the several AMF treatments, *Rhizophagus* sp. (56.59 g p-nitrophenol released h^−1^ g^−1^ soil) and *Funneliformis* sp. (31.99 g p-nitrophenol released h^−1^ g^−1^ soil) showed the highest levels of both acid (Figure 6a) and alkaline (Figure 6b) phosphatase activity in CR Dhan 207. Irrespective of the treatments, all rice varieties showed significantly higher acid and alkaline phosphatase activity in AMF-inoculated treatments as compared to the uninoculated control.

**Figure 7:**
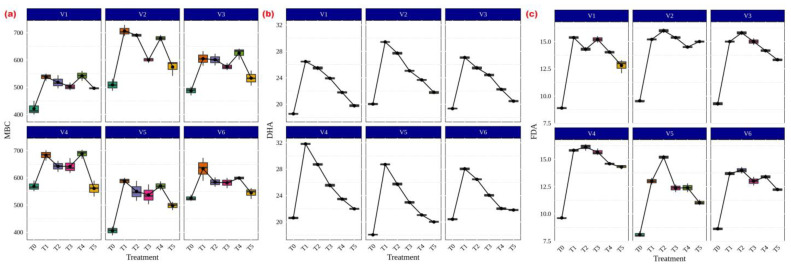
AMF and its influence on enhancement of microbial properties in different aerobic rice varieties. Abbreviations: (**a**) microbial biomass carbon (MBC) [µg g^−1^ soil]; (**b**) dehydrogenase activity (DHA) [µgTPF h^−1^g^−1^ soil]; (**c**) fluorescein diacetate assay (FDA) [µg fluorescein h^−1^g^−1^ soil].

In terms of microbial properties, *Funneliformis* sp., *Rhizophagus* sp., *Glomus* sp., *Acaulospora* sp. and *Claroideoglomus* sp. treatments significantly increased MBC in CR Dhan 204 and CR Dhan 207 (706.8 and 688.4 µg g^−1^ soil) (Figure 7a). A similar trend was also noticed in DHA (29.43 and 31.82 µgTPF h^−1^g^−1^ soil) (Figure 7b) and FDA (15.37 and 16.13 µg fluorescein h^−1^g^−1^ soil) (Figure 7c).

Through increasing microbial activity in the soil or by the exudation of enzymes by plants, AMF can also have an impact on soil enzyme activity as well as plant growth promotion [53,54,55]. Several studies have described how AMF intervention could stimulate soil enzyme activity through soil microorganisms [20,27,56,57]. Generally, soil enzymes are primarily produced by microorganisms; others, such as phosphatase [58], urease, and peroxidases, are also secreted by plant roots. Reports [59,60,61] have shown that the effects of AMF on various soil enzyme activities and growth-promoting compounds, which release the more biologically accessible nutrients from complex materials, were positively correlated with increasing ratios of soil-available P and plant biomass as well as strongly abiotic context-dependent factors, with beneficial implications for plant growth. All of the aforementioned data made it very evident that AMF will increase soil enzyme activity, which could improve nutrient cycling.

### 3.6. Assessing the Mycorrhizal Responsiveness in Different Aerobic Rice Varieties

**Figure 8:**
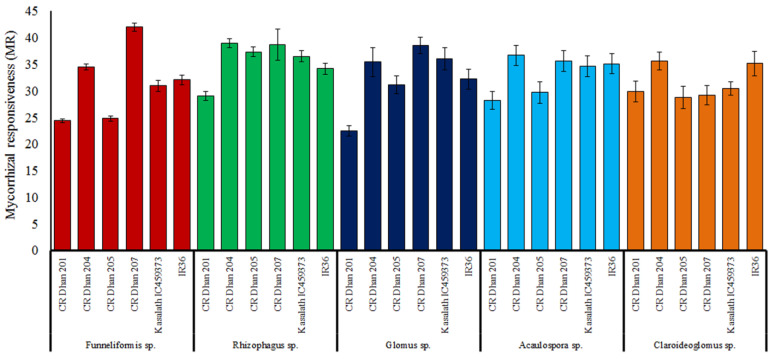
Mycorrhizal responsiveness in six aerobic rice varieties with five AMF inoculum effects.

Out of the selected rice varieties, mycorrhizal responsiveness was found highest in CR Dhan 207 followed by CR Dhan 204, CR Dhan 205 and Kasalath IC459373 with the application of *Funneliformis* sp. and *Rhizophagus* sp. under P-deficient conditions (Figure 8); however, the AMF responsiveness varies with different rice varieties.

### 3.7. Correlation of AMF Colonization with Soil and Plant Properties Using Linear Models

The linear model was used to select the important parameters linked to AMF colonization and to calculate the correlation of the important variables (Table 2).

**Figure 9:**
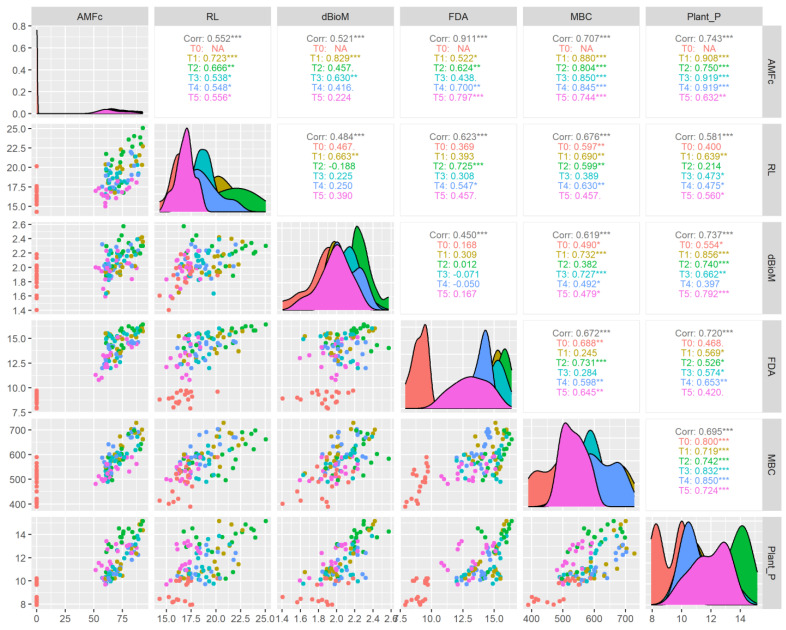
Correlation of AMF treatments in different aerobic rice varieties on plant P uptake and soil microbial properties. * *p* < 0.05, ** *p* < 0.01, *** *p* < 0.001.

The correlation analysis (Figure 9) showed that AMF colonization had a significant (*p* < 0.001) positive correlation with FDA (R^2^ = 0.911), MBC (R^2^ = 0.707) and plant-available P (R^2^ = 0.743). The correlation between AMF colonization and FDA, the *Claroideoglomus* sp. (R^2^ = 0.797) and *Acaulospora* sp. (R^2^ = 0.700) treatments, showed a higher coefficient than other treatments. Similarly, with AMF colonization and MBC correlation, the higher coefficients were recorded in the treatment *Funneliformis* sp. (R^2^ = 0.880) followed by *Glomus* sp. (R^2^ = 0.850), *Acaulospora* sp. (R^2^ = 0.845), —*Rhizophagus* sp. (R^2^ = 0.804) and *Claroideoglomus* sp. (R^2^ = 0.744) at *p* < 0.011 levels of significance. The correlation coefficient between AMF colonization and plant P was significantly (*p* < 0.01) at par for microbial treatments *Acaulospora* sp. (R^2^ = 0.919), *Glomus* sp. (R^2^ = 0.919), *Funneliformis* sp. (R^2^ = 0.908), *Rhizophagus* sp. (R^2^ = 0.705), and *Claroideoglomus* sp. (R^2^ = 0.632). Similarly, many scientific reports have well documented that AMF plays a crucial role in soils for improving microbial activity, nutrient cycling, soil structure and plant–soil microbe interactions [62,63,64,65,66].

**Figure 10:**
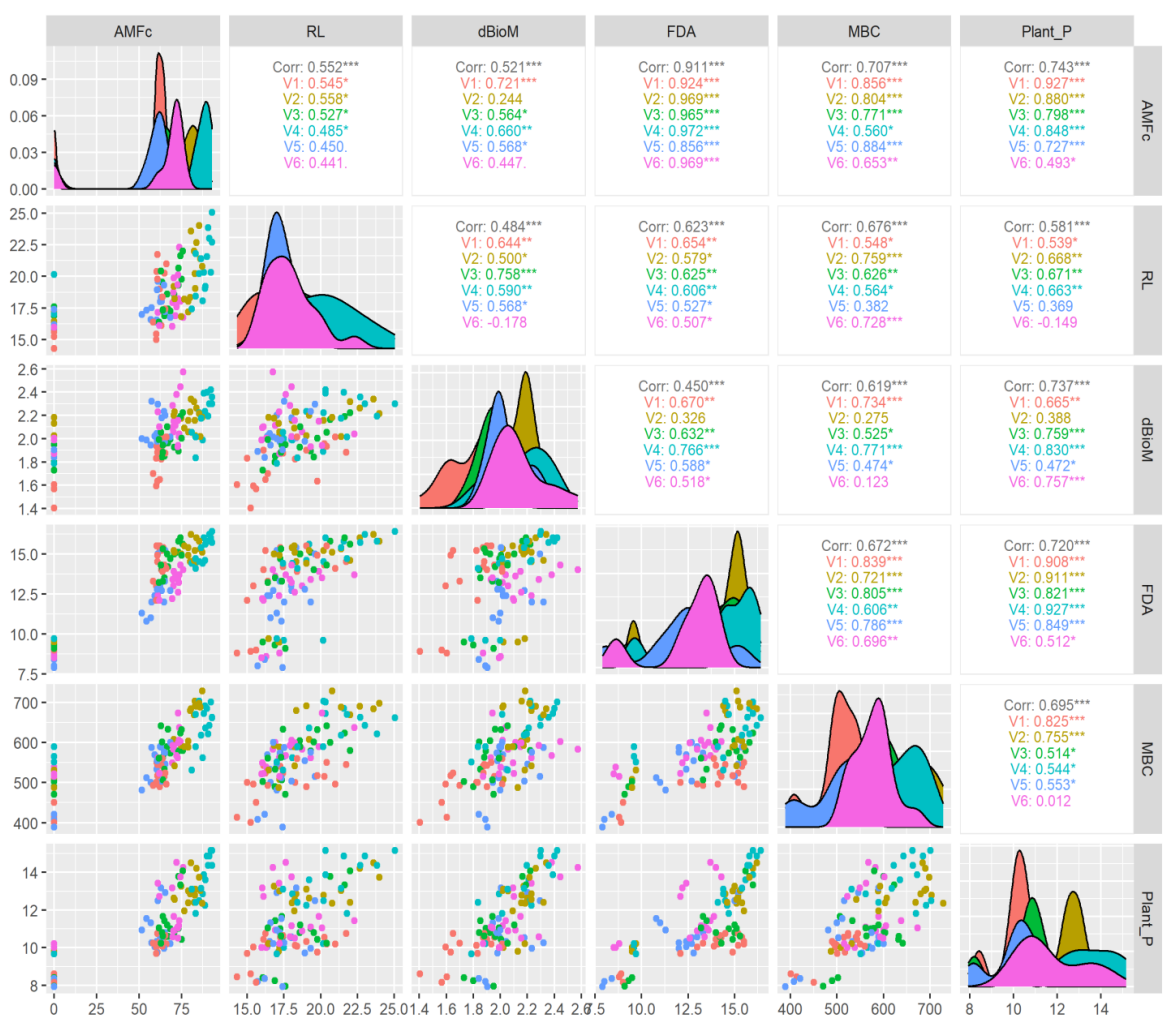
Response of aerobic rice varieties in AMF colonization correlation with plant P and soil microbial properties using Pearson correlation. * *p* < 0.05, ** *p* < 0.01, *** *p* < 0.001.

Further correlation studies among varieties given in Figure 10 show that CR Dhan 207 (R^2^ = 0.972), CR Dhan 204 (R^2^ = 0.969), and Kasalath IC459373 (R^2^ = 0.969) had the maximum coefficients between AMF colonization and FDA (R^2^ = 0.911) among the different aerobic varieties. However, the correlation between AMF colonization and MBC (R^2^ = 0.707) indicated that, among the varieties, IR 36 (R^2^ = 0.884) and CR Dhan 201 (R^2^ = 0.856) had the highest coefficient values, whereas CR Dhan 207 (R^2^ = 0.560) and Kasalath IC459373 (R^2^ = 0.653) registered the lowest coefficient among other varieties. Regarding the correlation between varieties and plant P uptake (R^2^ = 0.743), the highest coefficient was found in CR Dhan 207 (R^2^ = 0.927), at *p* < 0.001 significance. This finding clearly indicates that the response of AMF differs based on the type of variety. Thus, the selection of the right type of AMF is essential for exploring the maximum benefit from AMF symbiosis. Das et al. [67] reported that the application of *Glomus* spp. inoculation improved rice crop yields with better P availability in the rhizosphere under alternate wetting and drying irrigation.

## 4. Conclusions

Soil phosphorus deficiency is one of the major problems in aerobic rice cultivation. The fixation of this element in the soil makes it unavailable for plant uptake. The present study revealed that AMF intervention could significantly increase the plant growth and enhance P uptake by 16.60–28.50% compared to the control. Among the four different aerobic rice varieties, the mycorrhizal responsiveness was found to be superior in CR Dhan 207, followed by CR Dhan 204, CR Dhan 205, and CR Dhan 201. The linear modelling approach found that the AMF colonization in all the rice varieties had significant (*p* < 0.001) positive correlation with FDA, MBC, and P uptake, deciphering the importance of AMF association in rice for the improvement of phosphate availability to plants. The present findings require further field validation. However, results suggest that the external application of suitable AMF is essential for improving the plant growth and enhancing the uptake of P in aerobic rice in P-deficient soil.

## Figures and Tables

**Table 1:** Initial soil properties of the experimental soil sample.

pH (1:2.5, Soil: Water Suspension)	Electrical Conductivity (dS/m)	Available Phosphorus (kg/ha)	Available Nitrogen (kg/ha)	Available Potassium (kg/ha)
6.53 ± 0.06	0.48 ± 0.03	6.003 ± 0.59	236.75 ± 3.65	136.86 ± 3.97

**Table 2:** Identification of important parameters using step regression model.

Step	Variable	R-Square	Adj. R-Square	C(p)	AIC	RMSE
1	FDA	0.8292	0.8275	22.3958	844.7126	11.862
2	MBC	0.8459	0.8429	12.0246	835.5882	11.320
3	RL	0.8532	0.8490	8.5771	832.3012	11.0993
4	Plant P	0.8608	0.8554	5.0000	828.6062	10.8628

C(p): Mallows’ Cp constant; AIC: Akaike information criterion; RMSE: root mean square error.

## Data Availability

The data used to support the findings of this study are available from the corresponding author upon request.
